# Supplementary material for: Essential role for HSP40 in asexual replication and thermotolerance of malaria parasites
Source: bioRxiv. 2024 Nov 5:2024.11.05.622024. Preprint. [Version 1] doi: 10.1101/2024.11.05.622024 (PMC11580877; doi:10.1101/2024.11.05.622024)
Supplement: Supplement 1 [file NIHPP2024.11.05.622024v1-supplement-1.pdf]

bioRxiv preprint doi: https://doi.org/10.1101/2024.11.05.622024; this version posted November 5, 2024. The copyright holder for this preprint (which was not certified by peer review) is the author/funder, who has granted bioRxiv a license to display the preprint in perpetuity. It is made available under aCC-BY 4.0 International license.

Supporting Information

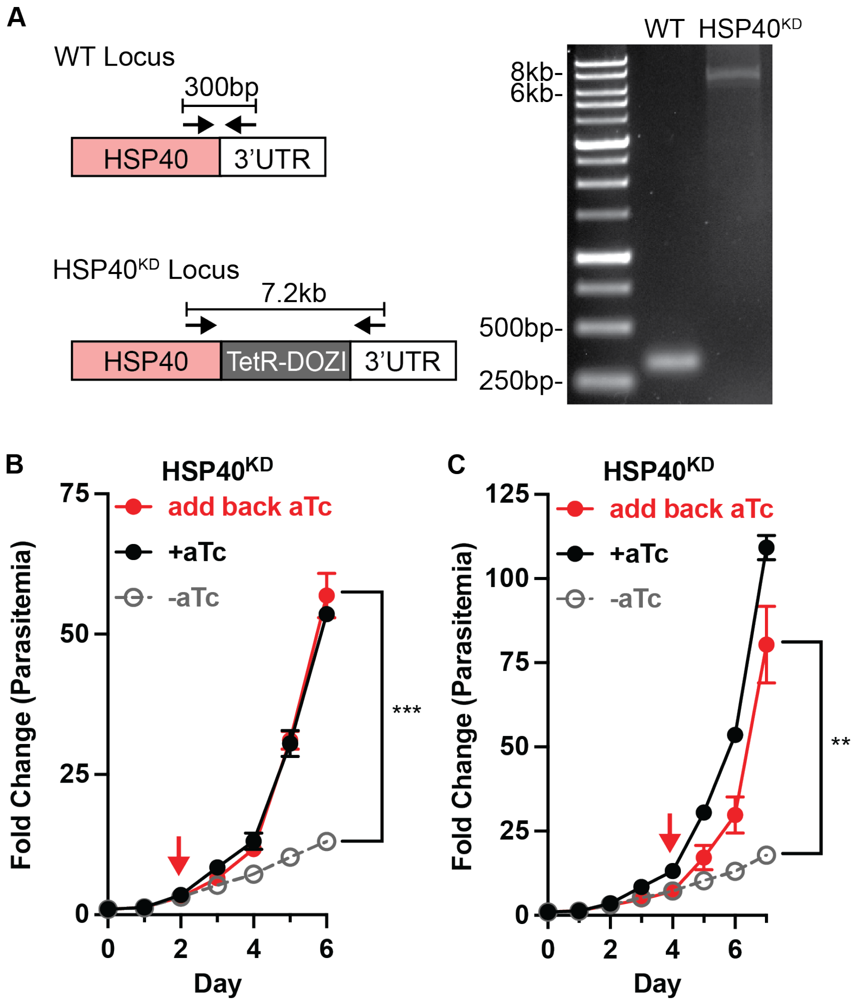

S1 Fig. HSP40^{KD} parasites demonstrate a reversible knockdown phenotype

A) PCR tests confirm genomic integration of the TetR-DOZI cassette at the HSP40 locus in *P. falciparum.* The same primer set (indicated by black arrows) was used for PCR with 3D7 (WT) and HSP40^{KD} genomic DNA. Growth assays of asynchronous HSP40^{KD} parasites measuring fold change in parasitemia by flow cytometry every 24 hours cultured +/- aTc, adding back aTc on either B) Day 2 or C) Day 4 -aTc (indicated by red arrow). Parasites were split 1:6 after day 4. Data represents the mean +/- SEM of three

bioRxiv preprint doi: https://doi.org/10.1101/2024.11.05.622024; this version posted November 5, 2024. The copyright holder for this preprint (which was not certified by peer review) is the author/funder, who has granted bioRxiv a license to display the preprint in perpetuity. It is made available under aCC-BY 4.0 International license.

biological replicates, missing error bars are too small to be visualized. Parametric unpaired t-tests between the add back aTc and -aTc condition were performed for the final day of collection (**p<0.01, *** p<0.001).

bioRxiv preprint doi: https://doi.org/10.1101/2024.11.05.622024; this version posted November 5, 2024. The copyright holder for this preprint (which was not certified by peer review) is the author/funder, who has granted bioRxiv a license to display the preprint in perpetuity. It is made available under aCC-BY 4.0 International license.

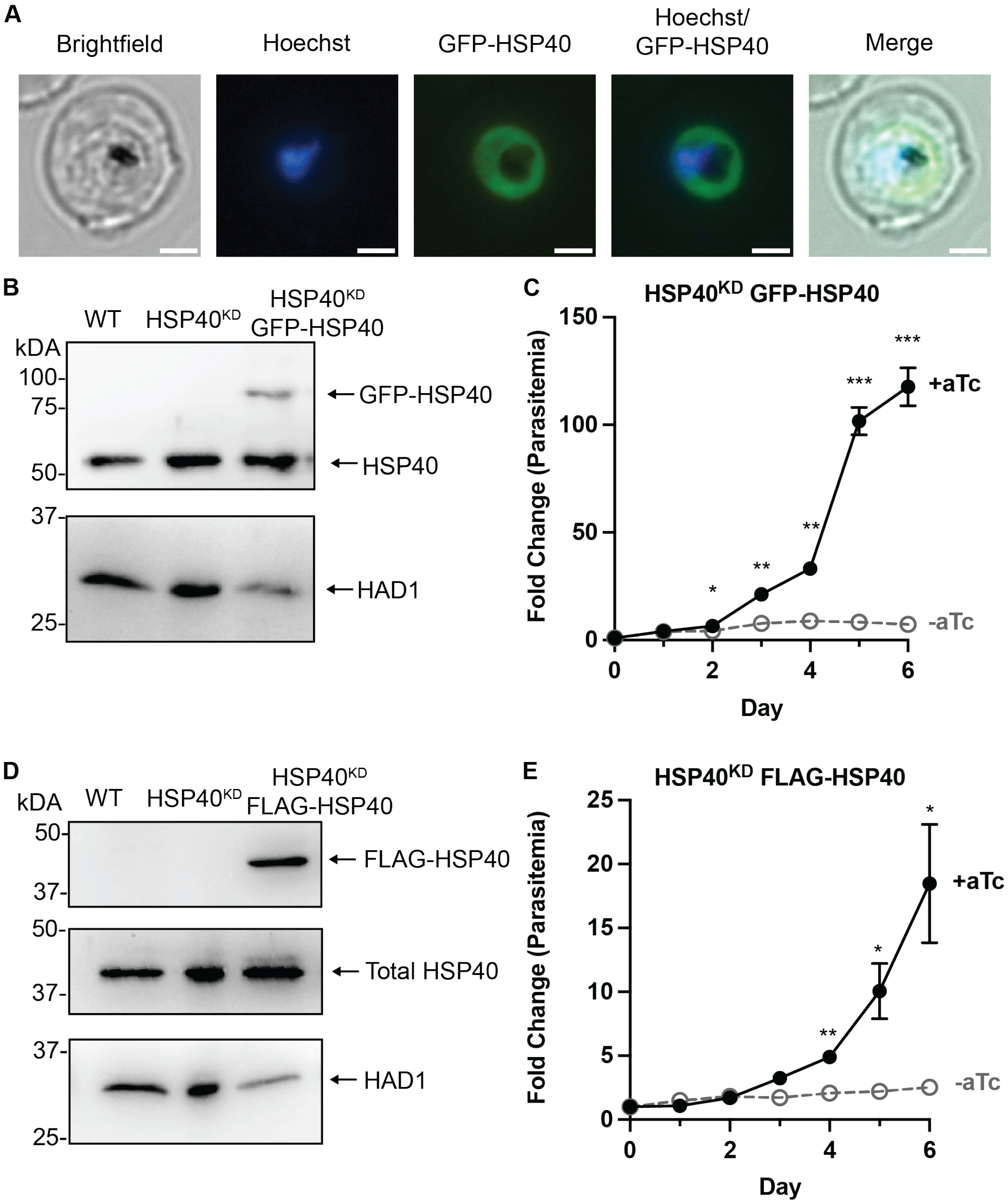

834

835 **S2 Fig. The function of HSP40 is disrupted by N-terminal tagging**

836 A) Representative fluorescence microscopy image shows HSP40^{KD} GFP-HSP40

837 parasites express GFP-HSP40, scale bar represents 2 microns. B) Representative anti-

838 HSP40 western blots of parasite lysates collected from HSP40^{KD} GFP-HSP40 parasites

bioRxiv preprint doi: https://doi.org/10.1101/2024.11.05.622024; this version posted November 5, 2024. The copyright holder for this preprint (which was not certified by peer review) is the author/funder, who has granted bioRxiv a license to display the preprint in perpetuity. It is made available under aCC-BY 4.0 International license.

show expression of the GFP-HSP40 fusion protein. HAD1 was used as a loading control. Blot is representative of three biological replicates. C) Growth assay of asynchronous HSP40^{KD} GFP-HSP40 parasites measuring parasitemia by flow cytometry every 24 hours cultured +/- aTc. Parasites were split 1:6 after day 2. Data represents the mean +/- SEM of three biological replicates, missing error bars are too small to be visualized. Parametric unpaired t-tests were performed (*p<0.05, **p<0.01, *** p<0.001). D) Representative anti-FLAG and anti-HPS40 western blots of parasite lysates collected from HSP40^{KD} FLAG-HSP40 parasites show the complement stain expresses FLAG-HSP40. HAD1 was used as a loading control. Blot is representative of three biological replicates. E) Growth assay of asynchronous HSP40^{KD} FLAG-HSP40 parasites measuring parasitemia by flow cytometry every 24 hours cultured +/- aTc. Parasites were split 1:6 after day 4 collection. Data represents the mean +/- SEM of three biological replicates, missing error bars are too small to be visualized. Parametric unpaired t-tests were performed (*p<0.05, **p<0.01).

bioRxiv preprint doi: https://doi.org/10.1101/2024.11.05.622024; this version posted November 5, 2024. The copyright holder for this preprint (which was not certified by peer review) is the author/funder, who has granted bioRxiv a license to display the preprint in perpetuity. It is made available under aCC-BY 4.0 International license.

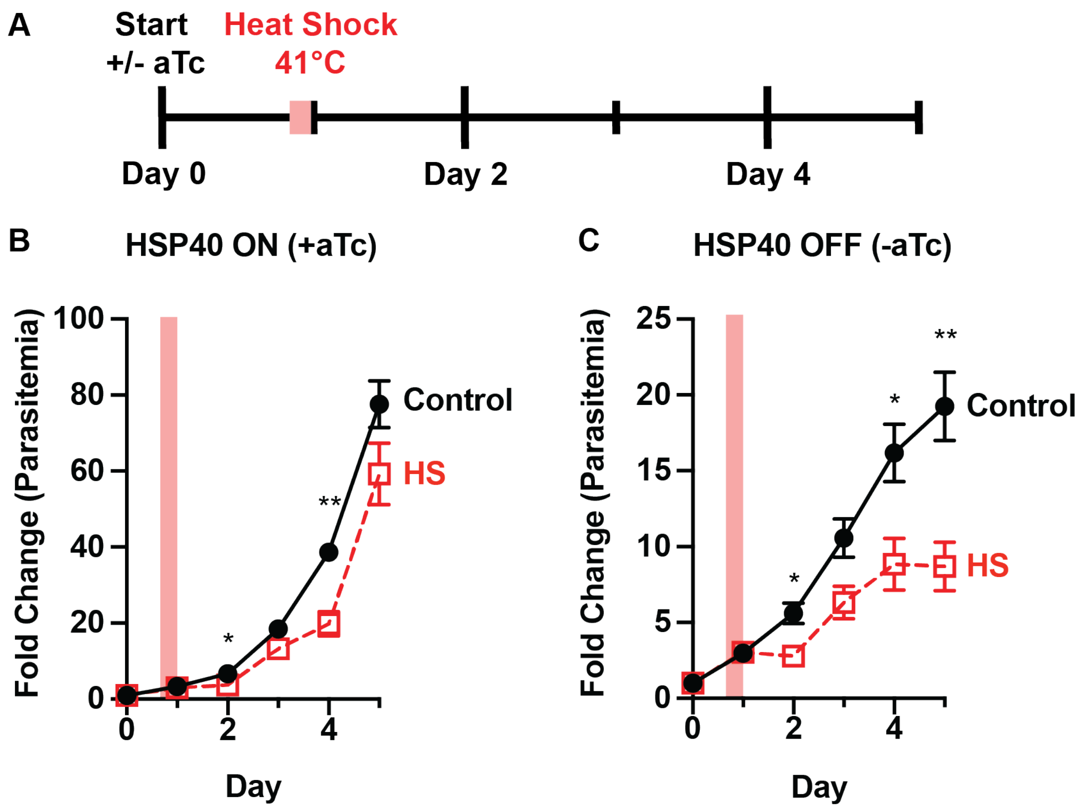

S3 Fig. Heat shock after one day of HSP40 knockdown shows reduced parasite thermotolerance.

A) Experimental design to assay thermotolerance: HSP40^{KD} parasites were subjected to a 6-hour 41°C heat shock (HS) on day one +/- aTc. Parasitemia was measured by flow cytometry collecting every 24 hours. Cultures were split 1:4 after day 3 collection. Growth assays measuring HSP40^{KD} after a 6hr heat shock (HS) show parasites recover from heat shock when B) HSP40 expression is on but lose this ability when C) HSP40 expression is off. Data represents the mean +/-SEM of biological replicates, missing error bars are too small to visualize. Parametric unpaired t-tests were performed (*p<0.05, **p<0.01).

bioRxiv preprint doi: https://doi.org/10.1101/2024.11.05.622024; this version posted November 5, 2024. The copyright holder for this preprint (which was not certified by peer review) is the author/funder, who has granted bioRxiv a license to display the preprint in perpetuity. It is made available under aCC-BY 4.0 International license.

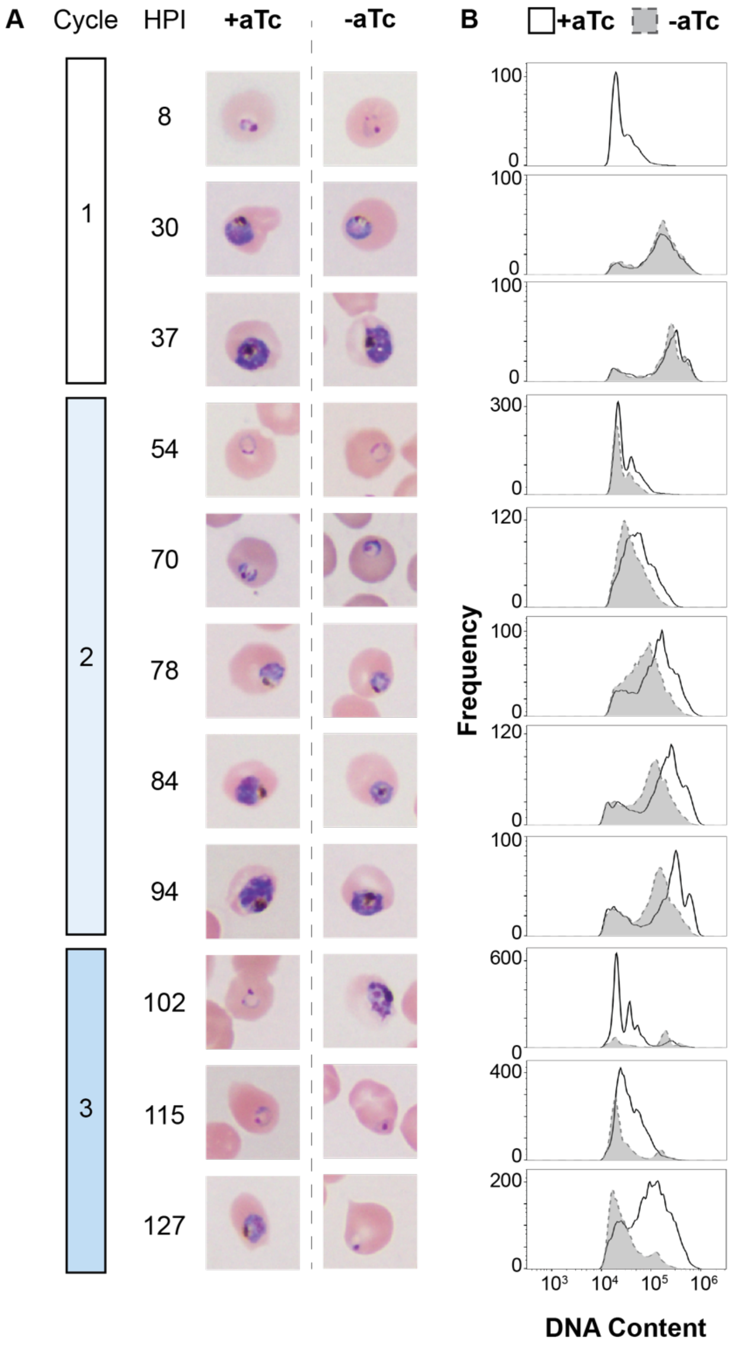

S4 Fig. HSP40^{KD} parasites demonstrate a second cycle developmental defect

A) Tightly synchronized HSP40^{KD} parasites were monitored for lifecycle progression starting +/-aTc at 8 hours post invasion (HPI) through the third cycle of replication. During Cycle 2, there is a developmental lag starting when +aTc is 84hpi and continues

bioRxiv preprint doi: https://doi.org/10.1101/2024.11.05.622024; this version posted November 5, 2024. The copyright holder for this preprint (which was not certified by peer review) is the author/funder, who has granted bioRxiv a license to display the preprint in perpetuity. It is made available under aCC-BY 4.0 International license.

870 as +aTc parasites enter cycle 3. Data is representative of 3 biological replicates. B)
871 Histograms of infected red blood cell DNA content in HSP40^{KD} parasites +/- aTc from
872 flow cytometry samples collected at time points indicated in part A. Starting at 84 HPI
873 when the +aTc condition progresses into schizogony and increases the DNA content of
874 cells, the -aTc condition lags. Entering cycle 3 at 102 HPI, the +aTc condition shows a
875 large population with predominantly lower DNA content due to the newly invaded cycle
876 3 rings, while the -aTc has a smaller total population of cells with higher DNA content.
877 Data is representative of 3 biological replicates.

bioRxiv preprint doi: https://doi.org/10.1101/2024.11.05.622024; this version posted November 5, 2024. The copyright holder for this preprint (which was not certified by peer review) is the author/funder, who has granted bioRxiv a license to display the preprint in perpetuity. It is made available under aCC-BY 4.0 International license.

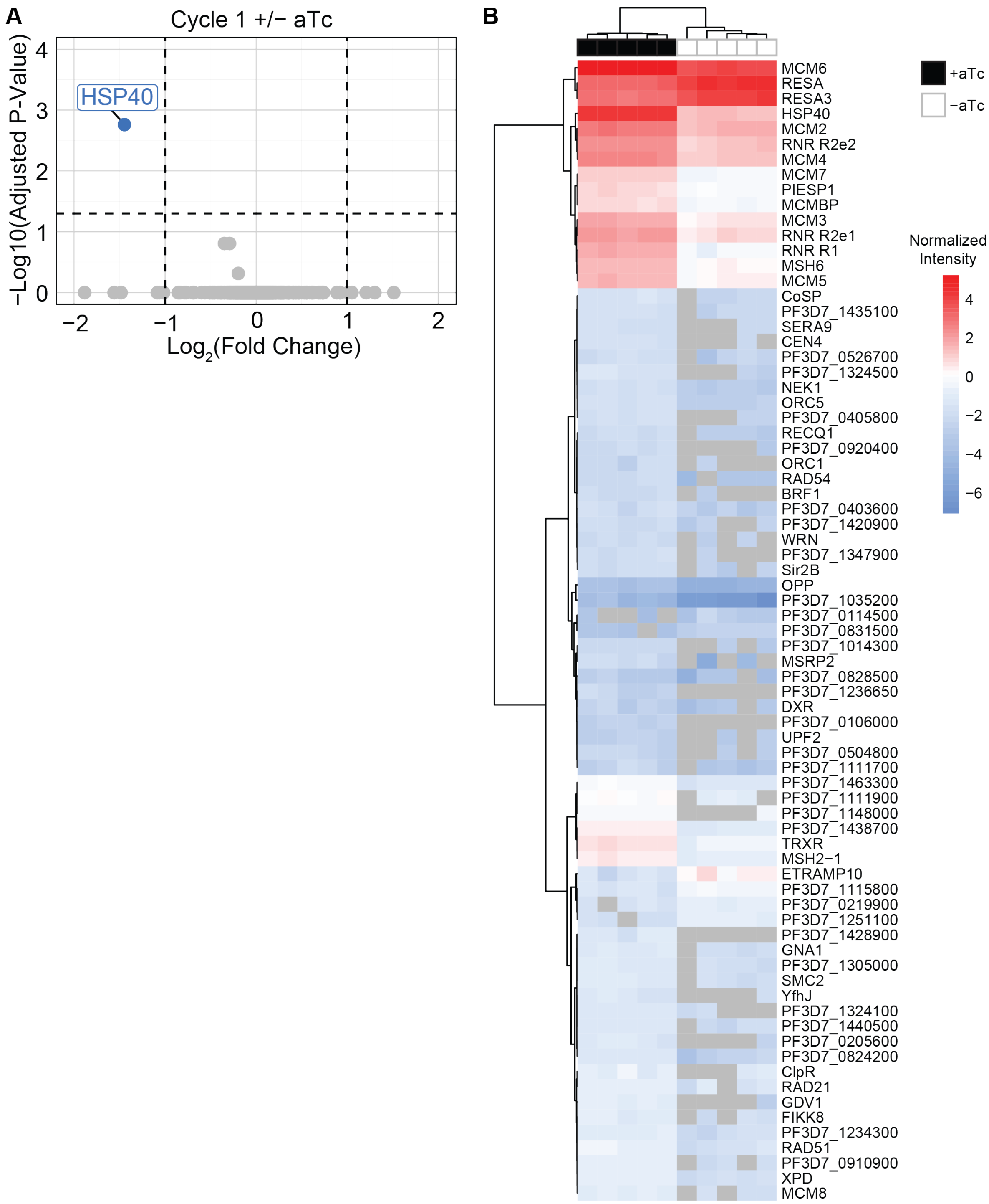

S5 Fig. Whole-Cell Proteomics of HSP40^{KD} parasites +/-aTc

A) Volcano plot of cycle 1 +/- aTc differential abundance analysis, HSP40 was the only protein with significantly different expression. B) Heat map of the normalized intensity

bioRxiv preprint doi: https://doi.org/10.1101/2024.11.05.622024; this version posted November 5, 2024. The copyright holder for this preprint (which was not certified by peer review) is the author/funder, who has granted bioRxiv a license to display the preprint in perpetuity. It is made available under aCC-BY 4.0 International license.

of all 75 differentially expressed proteins cycle 2 +/- aTc across N=5 biological replicates detected by proteomics. Hierarchical clustering was performed using Euclidean distance and Ward method for columns and rows. Peptides that were not detected are NA in grey.
